# Supplementary material for: SmdA is a Novel Cell Morphology Determinant in Staphylococcus aureus
Source: mBio. 2022 Mar 31;13(2):e03404-21. doi: 10.1128/mbio.03404-21 (PMC9040797; doi:10.1128/mbio.03404-21)
Supplement: FIG S1 [file mbio.03404-21-sf001.pdf]

```
Nosoc_WP_068130731          ----------------------------------MSTEILTEPLFIIVAALLLIVIISFL    26
Aliic_SEV88016              ----------------------------------MNTDIFLDPIFIAVAAVLLIVLTWFL    26
Salin_WP_020007446          ----------------------------------MNTEIFTNPIVIAILAVLLIVAVSFL    26
Jeotg_WP_026866661          ----------------------------------MNTEIFTNPIFIAIAVVLLIVVIWFL    26
Auric_AQL56528              ------------------------------MNINMNDFDMIHWIMFGLALLLLILLIFFI    30
Abyss_RPF57660              ------------------------------MNINMNDFDMIHWIMFGLALLLLILLVFFI    30
Mepid_RAK44338              --------------------------------MSVEVGSTLFYVLIGLGVLALLFFVLWL    28
Mcase_PKE19057              MQFYFLINLSKTMKLDMFFFIMKLLNIRKVRAMSLSNLQPMHYIVLGLAAALLLFIVLFI    60
Ssciu_WP_025905540          ----------------------------------MSQLEPIHYGLIAAIVIAILFIILFF    26
Sinte_PNZ51430              ----------------------------------MTQFGPMEIGLIAAIVVAALFFILFL    26
Schro_KDP13111              ----------------------------------MSQLDPIQIGLIAVSVLALLFLVLFL    26
Shyic_RTX86857              -------------------------------------MGPLEIGLVAAIVIAVIFLILFL    23
Saur_NCTC8325               ----------------------------------MDLSSPIVIGLIIAVVIALIFFVLFL    26
Ssimi_EHJ07687              ----------------------------------MDFSSPIVIGLIAAVVIALIFFVLYL    26
Swarn_PTI58951              ----------------------------------MSLSSPIGIGLIVAVVIAIIFFVLFL    26
Scap_TPX81490               ----------------------------------MSLSSPIVIGLIVAIIVAILFFILFL    26
Mabsc_SLD57873              ----------------------------------MDFSSPTVIGLIVAIPVAVLFFVLFL    26
Sepid_KAB2218069            ----------------------------------MDFSSPTVIGLIVAILVAVLFFILFL    26
Sarle_EJY95024              ----------------------------------MNSFGPIEIGLIVAVIVAVICLILFL    26
Ssapr_EHY92709              ----------------------------------MNSFGPIEIGLIVAIVVAVICLILFI    26
Scarn_RTX88654              ----------------------------------MNSFGPIEIGLIAAIVVAIICLILFL    26
Slugd_ADC87241              ----------------------------------MNSLGPMEIGLIVAVVIALICLILFF    26
                                                                        ..       :    ::

Nosoc_WP_068130731          IYYVKHRNEVEENERLYKKKEETLIESYVKNQEDERMAHKKEVSHLNEKYLEDTTLLNNK    86
Aliic_SEV88016              VYYFRSRNRVNKLTEEFDHEKQTLIEDYEATQEEDRLSHKKEVSGLNEKYNKDTEQLNKR    86
Salin_WP_020007446          VYFLKNRNRINTLTDEYSKEKEGLIEKYESNQEEERLNHKKEVSTLNEKYHTDTTLLNNK    86
Jeotg_WP_026866661          VYYFKHRNQVKVVENNHAKEKESLVQKYESEHEAERLEHKKELSNLNEKYYNDTTLLDNK    86
Auric_AQL56528              VKMIKANKQYKELQQARDDREKKLTSDYEKRIETERVDGKKKFSEQQSKYDAIVDDQSSQ    90
Abyss_RPF57660              VKMIKANKQYKELQQARDDREKKLTSDYEKRIETERVDGKKKFSEQQSKYDAIVDDQSSQ    90
Mepid_RAK44338              LALSSKKKSIARKEEEFRKAQKIRSDEYHDESEKSRLQYKKELAEQKETLQKTIDEKSSH    88
Mcase_PKE19057              YALSSKRKAIRAKEEALNKERSEMKSNYEESSEKSRLTFKKELAEQKDTYEAQLSTQNAQ    120
Ssciu_WP_025905540          VSLKQKQKSLNKIQEAHKKENETLKSEHKEKLDHERVENKKVLTKQEETHQEAISQKERE    86
Sinte_PNZ51430              VALKSKKKAKQTYATQYQTKQDKLTHEHQEELEKVRIDKKKAETRHKEEYETMVSSKNRE    86
Schro_KDP13111              FALRSKKKAKETYANQYQSRETKLNNEHKEALEKARIEKKKSDTRHKEEYDTMVSSKNRE    86
Shyic_RTX86857              TALNSKKKAQQQAEEQYEAKEKSLKDNYEDELEKERVEHKKTVTKQRADFDATVDSKDRE    83
Saur_NCTC8325               VALGSKKKVKRQTEEKYEQQEQNIKKSHEEALEKERIQNKKTITKQQEDYNHMVSTKDRE    86
Ssimi_EHJ07687              VALGSKKKVKRQTEEKYEQKEQNIKKTHEEQLEKERIENKKTITKQQEDYNEMVSTKDRE    86
Swarn_PTI58951              IALNSKKKIKQQTEEEYQQKEQSIKASHEEALEKERIENKKTVTKQKEDYEATVNSKERE    86
Scap_TPX81490               VANHSKKKIKNQTEAQYKEKEQHMKKSHEEALEKERVENKKAVTKQKEDFDATVSSKDRE    86
Mabsc_SLD57873              VANHSKKKVKNQTEAHYKEKEQHLKESHEEALEKERVENKKVVTKQQEDFDVTVSNKNRE    86
Sepid_KAB2218069            VANHSKKKVKNQTEAHYKEKEQHLKESHEEALEKERVKNKKVVTKQKEDFDVTVSNKNRE    86
Sarle_EJY95024              VTLKSKKKAQQKAEEHYQKKEKQLQDSYAEELEKERIENKKTVTKQKEDYDHTVNSKNRE    86
Ssapr_EHY92709              VALKSKKKAQEKVEAQYKSREQQLSDEHEEELEKERIENKKTVTKQKEEYTAAVNSKDRE    86
Scarn_RTX88654              VTLKSKNNIKQNTKEEYSLKEKQMLESHEEALEKERIENKKQVTRQKEDFDATISGKNRE    86
Slugd_ADC87241              VALRNNKKIKRQTVDEYKLKEKQMQESHDEALEKERIENKKTVTKQKENYEATVNSKERE    86
                                  .:            .      :    :  *:  **  :  .          . .

Nosoc_WP_068130731          LSSIQQFTVDKGEYLTDLALLNFKNKLVTEERIRESDMYILSNIYLPSRNYTNTRKIDHL    146
Aliic_SEV88016              LRSVSQFTSDKGEYLTDLALKDLKNQLVKEDKIRDLDMHILSNIYLPSRNYTNTRKVDHL    146
Salin_WP_020007446          LSSLRQFTVDKGEYLTDLSLIQLKERLVRDEKIRETDMHILSNVYLPSRNYTNTRKIDHL    146
Jeotg_WP_026866661          LSSLHQFSVDKGEYLTDLALIQLKDKLVKDEKIRESDMIILSNVFLPSRNYTNTRKIDHL    146
Auric_AQL56528              ISSLKQFTYGKSQYLTDITLLSFRDKLIDQERIRPEDMHVLANVLIPSKNYKQTKQVDHV    150
Abyss_RPF57660              ISSLKQFTYGKSQYLTDITLLSFRDKLIDQERIRPEDMHVLANVLIPSKNYKQTKQVDHV    150
Mepid_RAK44338              IESLKMFSKDKGEYLTDLTLIQLKEQFIREERIRPEDMHVLANIYIPGKRVKSTDKLDHV    148
Mcase_PKE19057              IDSLKLFSRDKGEYLTDLTLINLKDNLVAQERIRPEDMHVLANIYIPGKRVKSTDRLDHV    180
Ssciu_WP_025905540          IDSLKLFSKNEGEYITDRHLLELRDQLVNERRIRPEDMHIMANIFLPKDPLGKVRQIDHL    146
Sinte_PNZ51430              IDALKLFSKNHSEYVTDMRLLGIRERLVKEKRIRPEDMHIMANIFLPTNDLEDITRVSHL    146
Schro_KDP13111              IDALKLFSKNDSEYITDMRLLGIRERLVKEKRIRPEDMHIMANIFMPTNDLEEITRISHL    146
Shyic_RTX86857              IDALKLFSKNHSEYITDMRLIGIRERLVKEKRIRPEDMHIMANIFLPKNDMNDIERISHL    143
Saur_NCTC8325               IDALKLFSKNHSEYVTDMRLIGIRERLVKEKRIRPEDMHIMANIFLPKDGFNNIERISHL    146
Ssimi_EHJ07687              IDALKLFSKNHSEYVTDMRLIGIRERLVKEKRIRPEDMHIMANIFLPSNKFNDIERISHL    146
Swarn_PTI58951              IDALKLFSKNHSEYVTDLRLIGIRERLVNEKRIRPEDMHIMANIFLPTNELNKIERISHL    146
Scap_TPX81490               IDALKLFSKNHSEYVTDMRLIGIRERLVNEKRIRPEDMHIMANIFLPSNEFNNIERISHL    146
Mabsc_SLD57873              IDALKLFSKNHSEYVTDMRLIGIRERLVNEKRIRPEDMHIMANIFLPSNELTNIERVSHL    146
Sepid_KAB2218069            IDALKLFSKNHSEYVTDMRLIGIRERLVNEKRIRPEDMHIMANIFLPSNELTNIERVSHL    146
Sarle_EJY95024              IDALKLFSKNHSEYVTDMRLIGIRERLVNEKRIRPEDMHIMANIFLPRNEFSDVQRISHL    146
Ssapr_EHY92709              IDALKLFSKNQSEYVTDMRLIGIRERLVNEKRIRPEDMHIMANIFLPRNEFSDVQRISHL    146
Scarn_RTX88654              IDALKLFSKNTSEYVTDMRLIGIRDRLVNEKRIRRDDMHIMANIFLPSNEFNDIQRISHL    146
Slugd_ADC87241              IDALKLFSKNTSEYVTDMRLIGIRERLVNEKRIRPEDMHIMANIFLPGNDLNNIERISHL    146
                            : ::  *: . .:*:**  *  :::.:: : :**  ** :::*: :*     .  ::.*:
```

```
Nosoc_WP_068130731      VLTRTGIYLIDSKYWSGHILHGVTEEQYDEIPYLEGIFQLLNLDPNKEQTLIFEKEND--    204
Aliic_SEV88016          VLTRTGIYLMESRFWKGHIIHGVSEQNFEQLPYVENFFELLGLNKKQEQTFIFEKKDD--    204
Salin_WP_020007446      VLTRTGIYMIDSKYWRGHILHGINEENFEELPYTESFFDLLELDKTKEQTLIFEKSDS--    204
Jeotg_WP_026866661      VLTRTGIYLIDSKYWSGHILHGVNEAQFETVPYVESFFDLLDLDKKREQTLIFEKADQ--    204
Auric_AQL56528          ILTRTGIYIVDSNYFSGHVYHGMNEQQFDQFPFLEGVYDALGYDHKDEYSFIVEPKDN--    208
Abyss_RPF57660          ILTRTGIYIVDSNYFSGHVYHGMNEQQFDQFPFLEGVYDALGYDHKDEYSFIVEPKDN--    208
Mepid_RAK44338          VLTRTGIYLIDSNYWTGHIYHGVSEMQFAGEPMLEGVFNILELDPKLEQTIVLDKNKD--    206
Mcase_PKE19057          ILTRTGIYVIDSNYWTGHLYHGISEMQFDGEPIFETVFNILGLDPKSEQTICLDKAED--    238
Ssciu_WP_025905540      VLTRTGIYVIDSNLVSGHIYHGITEQQFSDFPVLGQVFETLDLNPNKEQTLLLEKQPN-N    205
Sinte_PNZ51430          VLTRTGLYVIDSELLKGHVYQGISQQQFADNPMMEQVFKTLNLSPQTPQTVVLDQSEAAQ    206
Schro_KDP13111          VLTRTGLYIIDSELLKGHVYQGVSQNQFRENPMMEHVFKTLHLDGQTPQTIVLDQKED-Q    205
Shyic_RTX86857          VLTRTGLYIIDSQLLKGHVYNGISGKQFAELPTIEQVFNVLNLDQRTPQTLVLDENDD-Q    202
Saur_NCTC8325           VLTRTGLYIIDSQLLKGHVYNGISGGQFKDLPPMEQVFDTLDLDKSRPQTIVMDQNDD-K    205
Ssimi_EHJ07687          VLTRTGLYIIDSQLLKGHVYNGISGGQFKDLPPMEQVFKTLDLDASRPQTIIMDQNDD-Q    205
Swarn_PTI58951          VLTRTGLYIIDSQLLKGHVYNGISGAQFSELPTMEQVFNTLELDKGTPQTLVLDQNSD-E    205
Scap_TPX81490           VLTRTGLYIIDSQLLKGHVYNGISGAQFKELPTMSQVFQTLDLDESTPQTLVLDQNED-Q    205
Mabsc_SLD57873          VLTRTGLYIIDSQLLKGHVYNGISGAQFKELPTMSQVFDTLDLDSSQPQTLVLDQNED-Q    205
Sepid_KAB2218069        VLTRTGLYIIDSQLLKGHVYNGISGAQFKELPTMSQVFDTLDLDSSQPQTLVLDQNED-Q    205
Sarle_EJY95024          VLTRTGLYIIDSQLLKGHVYNGISGAQFEETPMMSQVFDTLDLDKKDPQVLVLDQNDD-T    205
Ssapr_EHY92709          VLTRTGLYIIDSQLLKGHVYNGVSAAQFKEQPMMEQVFNTLDLDGQVPQTLVLDQNED-K    205
Scarn_RTX88654          VLTRTGLYIIDSQLLKGHVYNGISGNQFQELPMMEQVFNTLDLDPKSPQTLVLDQNDD-K    205
Slugd_ADC87241          VLTRTGLYIIDSKLLKGHVYNGISGNQFNELPMMQQVFSTLDLNAKAPHTLVLDQNDD-Q    205
                        :*****:*:::*.   **: :*:.  ::   *    .:. *  .      . .:

Nosoc_WP_068130731      EKVAVNYYNNTIEDTEMTAEKLRNVLKLQFDVVPIIYFNPKDNGGHTIMNYATNSNAKVI    264
Aliic_SEV88016          LNVTVSHFNHMIEEAKLTAEKLKNVLKLQFDVVPVVFFNPEDNGNYSIANYSNDPSVKVL    264
Salin_WP_020007446      KNVSVNHYNDTIDETKTTAEKLRNVLKLEYDIVPMIYFNPKDNGNYSITNYSADPSVKVL    264
Jeotg_WP_026866661      KNVSVNHYNGVIDETKITAEKLKNKLKLQYDIVPIIYFNPKDNGNYSITNYSEDPSIRVI    264
Auric_AQL56528          GEVVMHPLDHQIQDLKVTAEKIRNILKLQYPVKTIMFYNEQETKRNVSINYSTDKDVIVL    268
Abyss_RPF57660          GEVVMHPLDHQIQDLKVTAEKIRNILKLQYPVKTIMFYNEQETKRNVSINYSTDKDVIVL    268
Mepid_RAK44338          GNAEFRTYNHSIEALKLKAERLEKVFELPYPVTPIAYYNAEGVGSEHITNYSRDNGVKVI    266
Mcase_PKE19057          GSAQFKSYKHSIEELKLKAERLEKVFELPYPVTPIAYYNAEGVGSEHITNYSRDNSVKVI    298
Ssciu_WP_025905540      KTAAFHSYTDEVKEVDNTTEELQRQLELKYTPTPIIYFHPNEVSEATISNYSQDPNIKVL    265
Sinte_PNZ51430          NTLSVVDYTDRLAHVEALAVKLQKELDLKYTPTPILYFNPRHEGDVTISNYASHGVTKVL    266
Schro_KDP13111          QKLTVIDYTKHLDSVEKLATTLQQKLNLKYAPTPILYFNPRHEGDITISNYAPQSGTKVL    265
Shyic_RTX86857          QSATFVNYSKQLHYVEQLADALQRQLNLKYTPMALLYFNPKNEGAVTISNYAQNTNTKVL    262
Saur_NCTC8325           RSLSFVNYSDQIEAIKQLAEDLQKELGAKYTPTSILYFNLKNEGDVTISNYNQNSAVKVL    265
Ssimi_EHJ07687          KSLSFVNYSDKLESIKQLAEDLQKELGTKYTPTSILYFNPKNDGDVTISNYNQNAAVKVL    265
Swarn_PTI58951          KSLSFINYSDEIQQIQNVAGDLQSKLNTKYTPTSILYFNPKNEGDVTISNYAQNSNVKVL    265
Scap_TPX81490           QSLSFVNYSEKIKHIQNLAGDLQSELNTKYTPTSILYFNPKNDGDVTISNYNQASNVKVL    265
Mabsc_SLD57873          HSLSFVNYSDKIKHIEKLAGDLQNELNTKYTPTSILYFNPKKDNDVTISHYTQSSNVKVL    265
Sepid_KAB2218069        HSLSFVNYSDKIKHIEKLAGDLQNELNTKYTPTSILYFNPKKDNDVTISHYTQSSNVKVL    265
Sarle_EJY95024          QSLSVVNYSNHLEAVEKLAGDLQNELNLKYTPTTILYFNPKNDGGVTISNFAQTANSKVL    265
Ssapr_EHY92709          DALSFVNYTTHLNEIERLAGDIQTELNLKFTPTTILYFNPKNDGDVTITNYAQSSNSKVL    265
Scarn_RTX88654          SSLSFVNYSDQLEAIEKLATDLQTQLGTKYTPTSIMYFNPKHDGDVTISNYAQSSSVKVL    265
Slugd_ADC87241          SSLSFLDYSAQLAQIEKLATDLQTKLGLTYTPTSILYFNPKREGEVTISNYAQTSSAKVL    265
                            .      :   .  :  :.  :   :    : ::: .        ::       *:

Nosoc_WP_068130731      VGTEQLEDYFNKHVFQGRFQYTVKDLDEIAESLMRLNP    302
Aliic_SEV88016          IGNEELDEYFKKYVFHGRFQYTVKDLTDIVDQLKALNP    302
Salin_WP_020007446      VGEEELEAFFLKYVFHGRFQYTVKQLDEIAEEIFNLNP    302
Jeotg_WP_026866661      VGQEDLEAFFLKYVFHGRFQYTVKDLDEIADAILEQSL    302
Auric_AQL56528          MGKPELEEYFEKHVFHGRFEYTVEELEQIKQQLLEMNP    306
Abyss_RPF57660          MGKPELEEYFEKHVFHGRFEYTVEELEQIKQQLLEMNP    306
Mepid_RAK44338          VGEEELQTFFEKFVFHGRFQYKVEALENIMDQIEHLNP    304
Mcase_PKE19057          VGEKELEHFFEKFVFHGRFQYKVEELEEVRAAIEHLNP    336
Ssciu_WP_025905540      VGEKQLQHFFNKFVFHGRFQYSVEDLERIMDEIEKFNP    303
Sinte_PNZ51430          VGPEQLNEFFNRFVFHGRIQYDVNALQNIMDEIESFN-    303
Schro_KDP13111          VGPAQLDEFFNKFVFHGRIQYDVNDLQAIMDEIEAFN-    302
Shyic_RTX86857          VGAEQLDEYFNKFVFHGRIQYNVEDLARIMEEIESFN-    299
Saur_NCTC8325           VGAEQLDEFFNKFVFHGRIQYNVEDLQQMMDKIESFN-    302
Ssimi_EHJ07687          VGKEQLDEFFNKFVFHGRIQYNVDDLQNIMDKIESFN-    302
Swarn_PTI58951          VGPEQLDEFFNKFVFHGRIQYNVDDLQQIMEQIESFN-    302
Scap_TPX81490           VGPEQLDEFFNKFVFHGRIQYNVDELQTIMDKIESFN-    302
Mabsc_SLD57873          VGPEQLEEFFNKFVFHGRIQYNVDDLQDIMDKIESFN-    302
Sepid_KAB2218069        VGPEQLDEFFNKFVFHGRIQYNVDDLQDIMDKIESFN-    302
Sarle_EJY95024          VGPEQLDEYFNKFVFHGRIQYNVEDLQQVMDKIESFN-    302
Ssapr_EHY92709          VGPEQLDEFFNKFVFHGRIQYNVEDLQRVMDEIESFN-    302
Scarn_RTX88654          VGPEQLDEFFNKFVFHGRIQFNVEELQSIMDKIETFN-    302
Slugd_ADC87241          VGPEQLNEFFNKFVFHGRIQYNVDDLQEIMTKIESFN-    302
                        :*  :*: :* :.**:**::: *. *  :   :   .
```

Fig. S1. Multiple sequence alignment of SmdA from different staphylococcal species. Protein sequences were aligned with Clustal Omega (1). The blue shaded residues are predicted to be extracellular, the transmembrane domain is shaded in grey, and the predicted NERD domain is shaded in yellow. *S. aureus* NCTC8325-4 is highlighted in bold, and residues that were mutated are marked in green. The accession numbers of the sequences are indicated, and the first five letter in the sequence tags indicate the genus or species corresponding the to the sequences (for example, Nosoc; *Nococomiicoccus*, Aliic; *Aliicoccus*, Salin; *Salinicoccus*, Jeotg; *Jeotgalicoccus*, Auric; *Auricoccus*, Abyss; *Abyssicoccus*, Mepid; *Macrococcus epidermidis*, Mcase; *Macrococcus caseolyticus*, Ssciu; *Staphylococcus sciuri*, Sinte; *Staphylococcus intermedius*).
