# Supplementary material for: SmdA is a Novel Cell Morphology Determinant in Staphylococcus aureus
Source: mBio. 2022 Mar 31;13(2):e03404-21. doi: 10.1128/mbio.03404-21 (PMC9040797; doi:10.1128/mbio.03404-21)
Supplement: FIG S2 [file mbio.03404-21-sf002.pdf]

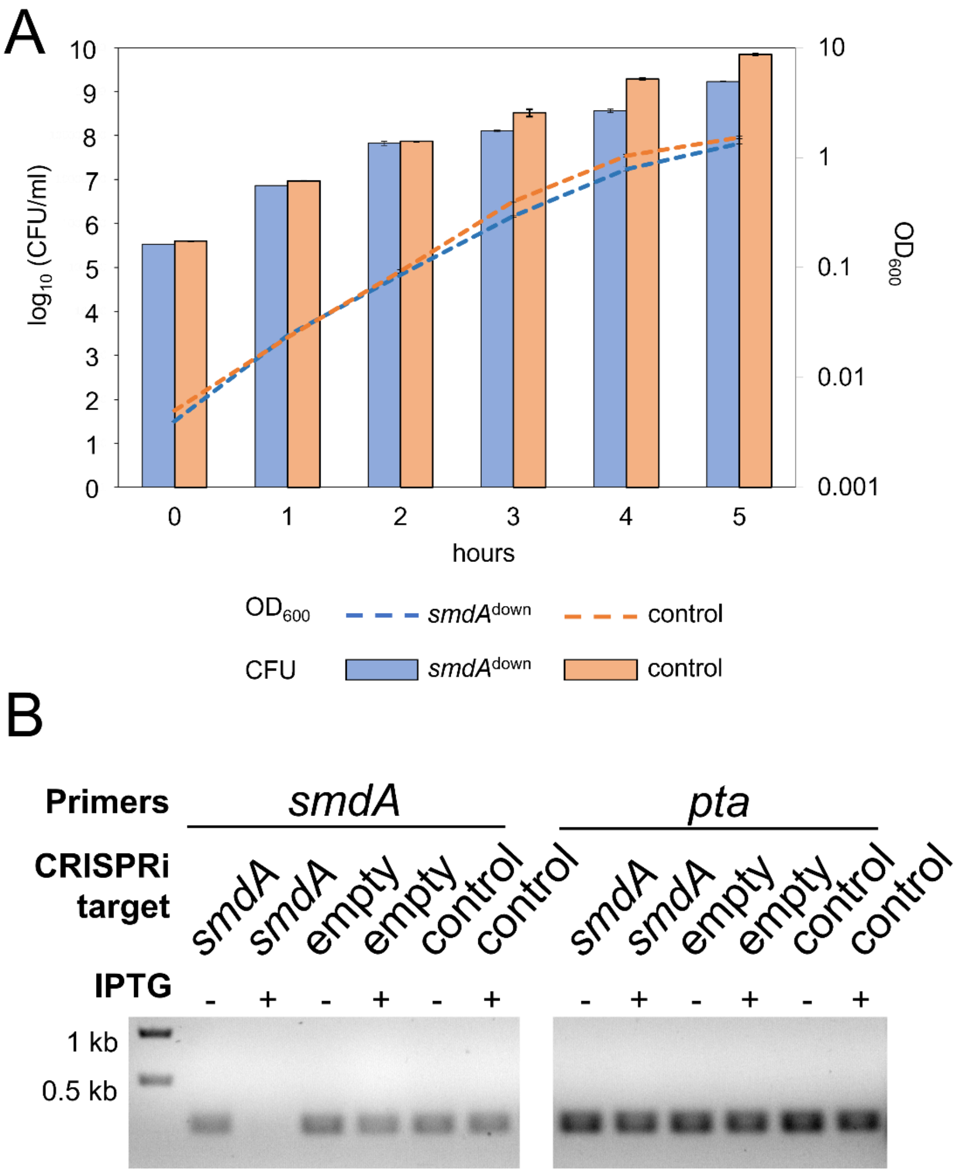

Fig. S2. Growth of SmdA knockdown strains in liquid cultures and verification of *smdA* silencing. (A) Growth of SmdA^{down} in *S. aureus* SH1000 (IM269) compared to the CRISRPi-control strain with a non-targeting sgRNA (IM284). IPTG (300 µg/ml) was added to induce expression of the CRISPRi-system and CFU/ml and OD_{600} measured every hour for five hours. (**B**) Verification of *smdA* silencing by PCR with RT-PCR. cDNA was synthesized from RNA isolated from induced and un-induced cultures of SH1000 SmdA^{down} (IM269) and the CRISPRi control strains (IM284; non-targeting sgRNA and IM165; empty plasmid without sgRNA). Primers targeting either *smdA* or the housekeeping gene *pta*.
