# Supplementary material for: SmdA is a Novel Cell Morphology Determinant in Staphylococcus aureus
Source: mBio. 2022 Mar 31;13(2):e03404-21. doi: 10.1128/mbio.03404-21 (PMC9040797; doi:10.1128/mbio.03404-21)
Supplement: FIG S3 [file mbio.03404-21-sf003.pdf]

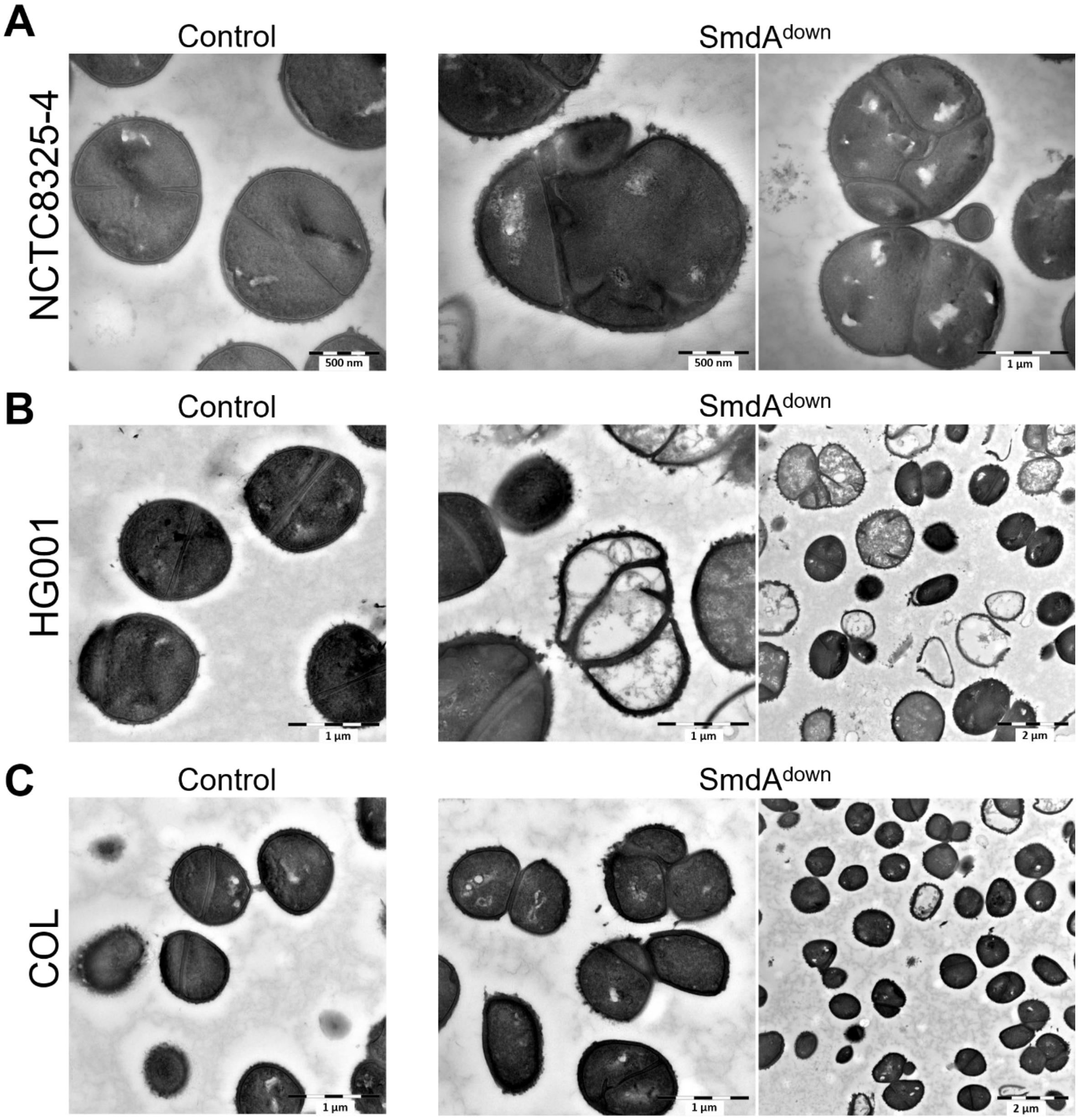

Fig. S3. Transmission electron microscopy (TEM) of different *S. aureus* strains with SmdA knockdown. SmdA^{down} and control cells analyzed with TEM in the *S. aureus* strains (**A**) NCTC8325-4 (IM311 and IM307), (**B**) HG001 (IM312 and IM313) and (**C**) COL (IM294 and IM295). The sizes of the scale bars are indicated in the images.
