# Supplementary material for: SmdA is a Novel Cell Morphology Determinant in Staphylococcus aureus
Source: mBio. 2022 Mar 31;13(2):e03404-21. doi: 10.1128/mbio.03404-21 (PMC9040797; doi:10.1128/mbio.03404-21)
Supplement: FIG S4 [file mbio.03404-21-sf004.pdf]

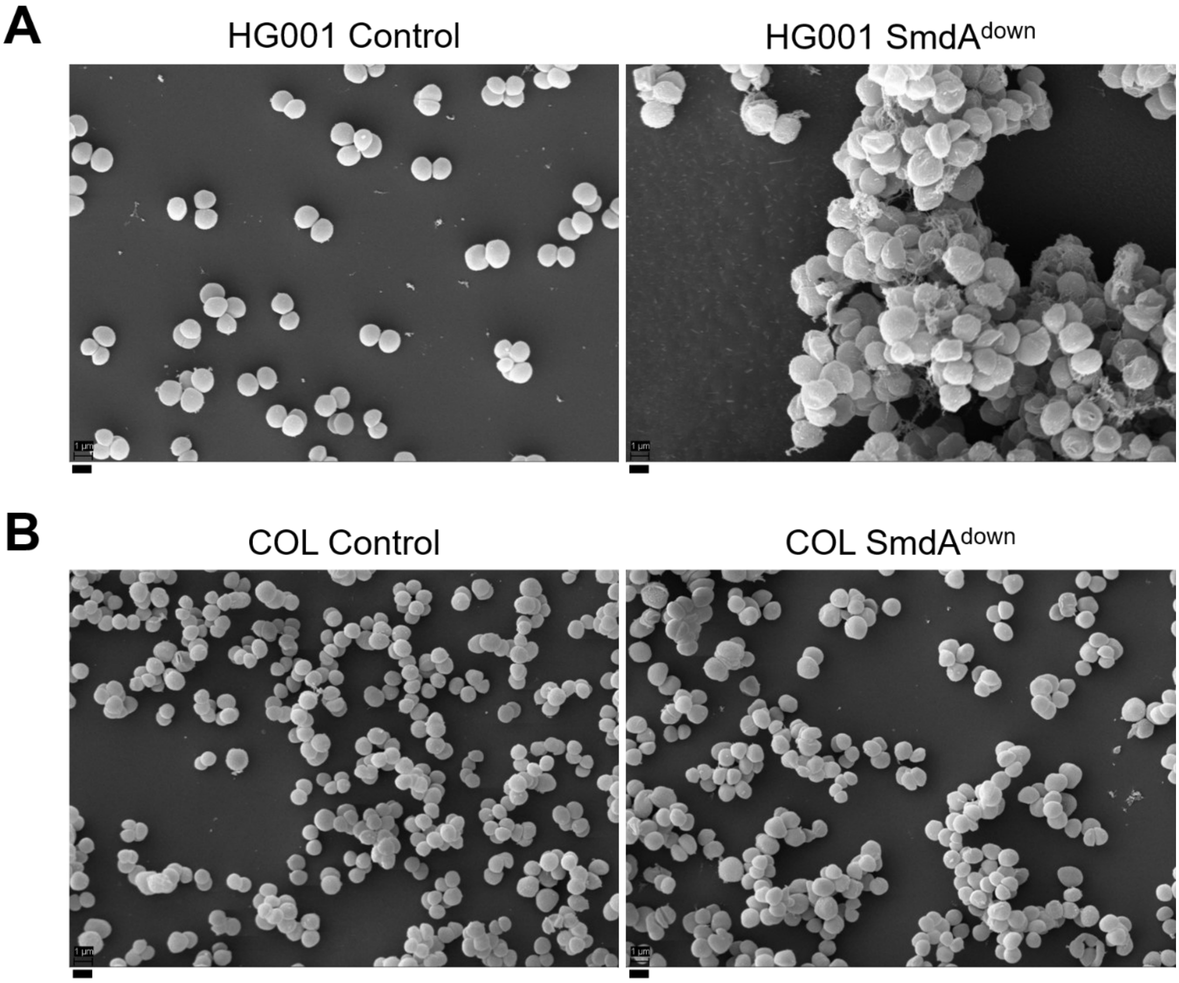

Fig. S4. Scanning electron microscopy (SEM) of cells depleted of SmdA. SEM micrographs of SmdA^{down} and control cells in *S. aureus* (**A**) HG001 (IM312 and IM313) and (**B**) COL (IM294 and IM295). All scale bars, 1 μm.
