# Supplementary material for: SmdA is a Novel Cell Morphology Determinant in Staphylococcus aureus
Source: mBio. 2022 Mar 31;13(2):e03404-21. doi: 10.1128/mbio.03404-21 (PMC9040797; doi:10.1128/mbio.03404-21)
Supplement: FIG S5 [file mbio.03404-21-sf005.pdf]

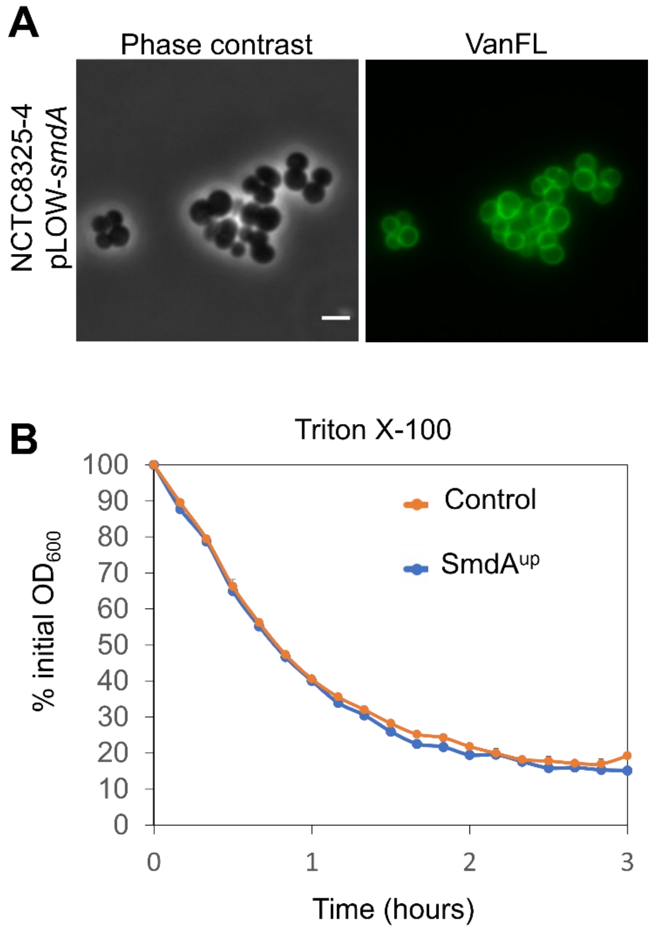

Fig. S5. Overexpression of SmdA. (**A**) Induced expression of an ectopic copy of *smdA* in the plasmid pLOW in *S. aureus* NCTC8325-4 (MK1866). Cells were labelled with the cell wall marker fluorescent vancomycin (VanFL). Scale bar, 2 µm. (**B**) Autolysis of *S. aureus* SmdA overexpression strain (MK1866) compared to plasmid control strain (MK1465) monitored in presence of 0.5 % Triton X-100. Results presented as % of initial OD_{600}. Error bars represent standard error calculated from four technical replicates.
