# Supplementary material for: SmdA is a Novel Cell Morphology Determinant in Staphylococcus aureus
Source: mBio. 2022 Mar 31;13(2):e03404-21. doi: 10.1128/mbio.03404-21 (PMC9040797; doi:10.1128/mbio.03404-21)
Supplement: FIG S6 [file mbio.03404-21-sf006.pdf]

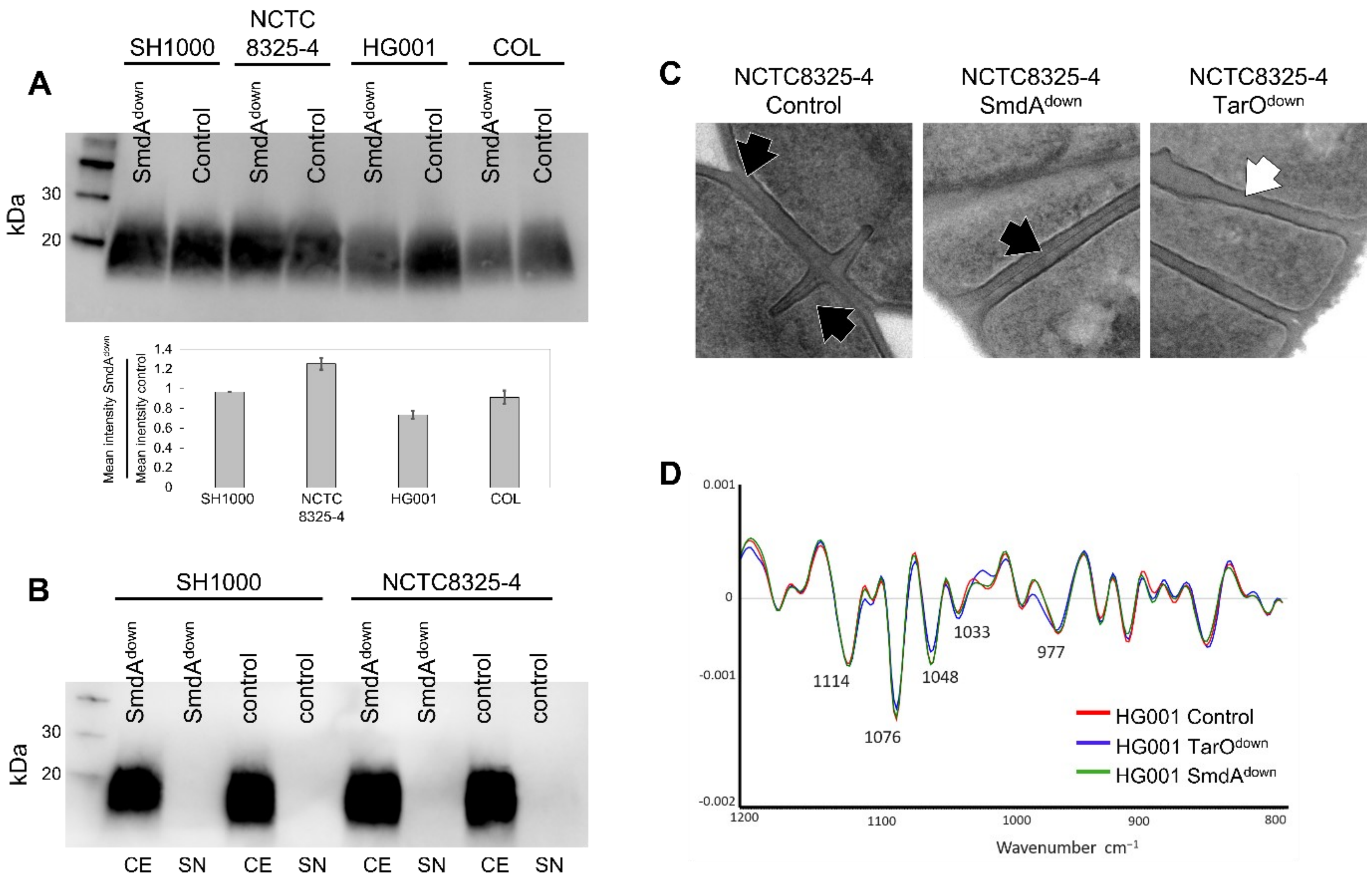
A
SH1000
NCTC 8325-4
HG001
COL
SmdA^down
Control
kDa
30
20
Mean intensity SmdA^down
Mean inentsity control
1.4
1.2
1
0.8
0.6
0.4
0.2
0
SH1000
NCTC 8325-4
HG001
COL
B
SH1000
NCTC8325-4
SmdA^down
control
CE
SN
C
NCTC8325-4 Control
NCTC8325-4 SmdA^down
NCTC8325-4 TarO^down
D
0.001
0
-0.001
-0.002
1114
1076
1048
1033
977
HG001 Control
HG001 TarO^down
HG001 SmdA^down
1200
1100
1000
900
800
Wavenumber cm^-1

Fig. S6. Analysis of teichoic acids. (**A-B**) Lipoteichoic acid (LTA) detection by immunoblotting with α-LTA antibody was performed with (**A**) cell extract samples from $SmdA^{down}$ and CRISPRi control cells from *S. aureus* SH1000 (IM269 and IM284), NCTC8325-4 (IM311 and IM307), HG001 (IM312 and IM313) and COL (IM294 and IM295), and (**B**) cell extract (CE)- and supernatant (SN) samples from $SmdA^{down}$ and CRISPRi control cells from *S. aureus* SH1000 and NCTC8325-4 (IM269, IM284, IM311 and IM307, respectively). In (A), the mean intensities in the bands (background subtracted) were determined using Fiji (2). The mean intensities of the LTA bands in the *smdA* depletions relative to their controls are plotted. All control strains express a non-targeting sgRNA. (**C-D**) SmdA does not have major impact on the synthesis of wall teichoic acids. (**C**) TEM micrographs of *S. aureus* NCTC8325-4 control strain (IM307) compared to SmdA and TarO knockdown strains IM311 and IM358, respectively. The black arrows indicate presence of a high-density layer in the septum, which is missing in the TarO depletion strain (white arrow). (**D**) Fourier transform infrared spectroscopy (FTIR) of *S. aureus* HG001 control strain (IM313) and knockdown strain of SmdA and TarO (IM312 and IM357, respectively). The polysaccharide region of the spectrum is shown, and the indicated peaks represents α- and β-glycosidic bonds.
