# Supplementary material for: SmdA is a Novel Cell Morphology Determinant in Staphylococcus aureus
Source: mBio. 2022 Mar 31;13(2):e03404-21. doi: 10.1128/mbio.03404-21 (PMC9040797; doi:10.1128/mbio.03404-21)
Supplement: TABLE S1 [file mbio.03404-21-st001.pdf]

Table S1. Proteins pulled down with SmdA-GFP.

**Protein**	**Description**	**Chromosomal *smdA-gfp*[a]**		**Plasmid-based *smdA-gfp***	
		Unique peptides	Ratio	Unique peptides	Ratio
**SmdA**	Uncharacterized protein	4	>4	35	8.8
**Atl**	Bifunctional autolysin	4	4	29	5.8
**SAOUHSC_01193**	Uncharacterized protein	3	>3	10	2.5
**FtsH**	ATP-dependent zinc metalloprotease	2	>2	22	11
**PrsA**	Foldase protein	2	>2	14	7
**SpsB**	Signal peptidase I	2	>2	12	>12
**FhuD2**	Heme ABC transporter	2	>2	12	>12
**Pbp2**	Penicillin-binding protein 2	1	>1	23	23
**SAOUHSC_01676**	UPF0365 protein	1	>1	14	14
**SAOUHSC_00356**	Uncharacterized protein	1	>1	14	>4
**FruA**	Fructose specific permease, putative	1	>1	12	>12
**AtpA**	ATP synthase subunit alpha	1	>1	12	>12
**AlaS**	Alanine--tRNA ligase	1	>1	12	12
**GlpK**	Glycerol kinase	1	>1	12	12
**NrdE**	Ribonucleoside-diphosphate reductase	1	>1	10	>10
RpoC	DNA-directed RNA polymerase subunit beta'	ND	NA	28	9.3
RpoB	DNA-directed RNA polymerase subunit beta	ND	NA	24	12
SAOUHSC_02525	Uncharacterized protein	ND	NA	18	>18
Pyk	Pyruvate kinase	ND	NA	17	5.7
AtpD	ATP synthase subunit beta	ND	NA	15	7.5
EzrA	Septation ring formation regulator	ND	NA	15	>15
GlpD	Aerobic glycerol-3-phosphate dehydrogenase	ND	NA	14	14
SAOUHSC_01895	Uncharacterized protein	ND	NA	14	>14
Pbp1	Penicillin-binding protein 1	ND	NA	14	>14
Pbp3	Penicillin-binding protein 3	ND	NA	14	>14
ClpB	Chaperone protein	ND	NA	14	>14
Mqo	Probable malate:quinone oxidoreductase	ND	NA	13	>13
SecD	Multifunctional fusion protein	ND	NA	13	>13
SAOUHSC_01974	Uncharacterized protein	ND	NA	13	>13
DltD	Protein DltD	ND	NA	12	>12
ClpC	ATP-dependent Clp protease ATP-binding subunit	ND	NA	12	>12
QoxA	Probable quinol oxidase subunit 2	ND	NA	11	11
GatB	Aspartyl/glutamyl-tRNA(Asn/Gln) amidotransferase	ND	NA	11	>11
SAOUHSC_00253	Uncharacterized protein	ND	NA	11	>11
SAOUHSC_00749	Uncharacterized protein	ND	NA	10	>10
Rny	Ribonuclease Y	ND	NA	10	>10
ArgG	Argininosuccinate synthase	ND	NA	10	>10
AccC	Biotin carboxylase	ND	NA	10	>10
HtrA1	Serine protease	ND	NA	10	>10

[a] ND; not detected, NA; not applicable.
