# Supplementary material for: SmdA is a Novel Cell Morphology Determinant in Staphylococcus aureus
Source: mBio. 2022 Mar 31;13(2):e03404-21. doi: 10.1128/mbio.03404-21 (PMC9040797; doi:10.1128/mbio.03404-21)
Supplement: FIG S7 [file mbio.03404-21-sf007.pdf]

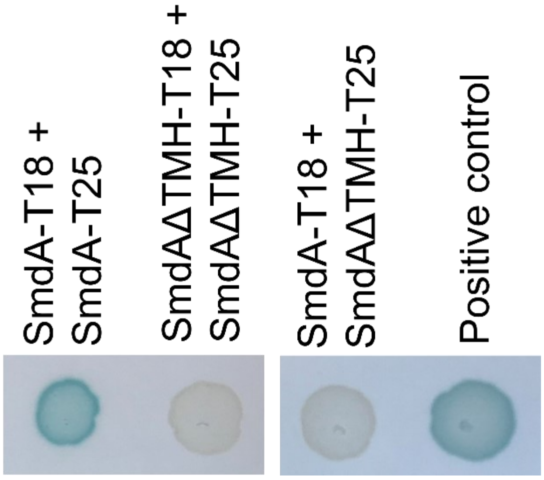

Fig. S7. Bacterial two-hybrid analysis demonstrating self-interaction between SmdA proteins. Blue bacterial spots indicate positive interactions and white spots indicate no interaction.
