# Supplementary material for: SmdA is a Novel Cell Morphology Determinant in Staphylococcus aureus
Source: mBio. 2022 Mar 31;13(2):e03404-21. doi: 10.1128/mbio.03404-21 (PMC9040797; doi:10.1128/mbio.03404-21)
Supplement: TABLE S2 [file mbio.03404-21-st002.pdf]

Table S2. Strains used in this study.

Name	Genotype[a]	Source
S. aureus		
NCTC8325-4	MSSA strain, derivative of NCTC8325, cured of phages.	(1)
SH1000	*rbsU*+ derivative of strain NCTC8325-4	(2)
RN4220	Restriction deficient derivative of NCTC8325-4	(3)
HG001	MSSA-strain, derivative of NCTC8325	(4)
COL	Hospital-associated MRSA strain	(5)
**CRISPRi depletion strains**		
SAMK13	SH1000, pLOW-*dcas9*	(6)
IM269	SAMK13, pCG248-sgRNA*(smdA*), ery^r, cam^r	This study
IM165	SH1000, pLOW-*dcas9*, pCG248(empty), ery^r, cam^r	This study
SAMK15/IM284	SAMK13, pCG248-sgRNA(control), ery^r, cam^r	(6)
IM307	NCTC8325-4, pLOW-*dcas9*_extra_*lacO*, pCG248-sgRNA(control), ery^r, cam^r	This study
IM311	NCTC8325-4, pLOW-*dcas9*, pCG248-sgRNA*(smdA*), ery^r, cam^r	This study
IM313	HG001, pLOW-*dcas9*, pCG248-sgRNA(control), ery^r, cam^r	This study
IM312	HG001, pLOW-*dcas9*, pCG248-sgRNA*(smdA*), ery^r, cam^r	This study
IM294	COL, pLOW-*dcas9_aad9*, pCG248-sgRNA*(smdA*), spc^r, cam^r	This study
IM295	COL, pLOW-*dcas9_aad9*, pCG248-sgRNA(control), spc^r, cam^r	This study
IM358	NCTC8325-4, pLOW-*dcas9*, pCG248-sgRNA*(tarO*), ery^r, cam^r	This study
IM357	HG001, pLOW-*dcas9*, pCG248-sgRNA*(tarO*), ery^r, cam^r	This study
IM293	SH1000, pLOW-*dcas9*_P*atl-luc*, pCG248-sgRNA(*walR*) , ery^r, cam^r	This study
**Strains for localization studies**		
IM104	SH1000, pLOW-*SAOUHSC_01908-m(sf)gfp,* ery^r	This study
IM305	NCTC8325-4, pLOW-*smdA-m(sf)gfp,* ery^r	This study
IM373	NCTC8325-4, pLOW-*smdAΔTMH-m(sf)gfp,* ery^r	This study
IM308	SH1000, *smdA-m(sf)gfp_aad9,* spc^r	This study
HC060	SH1000, pLOW-*smdA-mYFP*, pHC-*ftsZ-mKate2,* ery^r, neo^r	This study
SH4639	SH1000, *ezrA-gfp*, kan^r	(7)
MK1952	SH4639, pLOW-*dcas9*, pCG248-sgRNA(*smdA*), ery^r, cam^r	This study
MK1953	SH4639, pLOW-*dcas9*, pCG248-sgRNA(control), ery^r, cam^r	This study
**Strains used for overexpression and mutagenesis**		
MK1465	NCTC8325-4, pLOW-dcas9, ery^r	This study
MK1866	NCTC8325-4, pLOW-*smdA*, ery^r	This study
MK1911	NCTC8325-4, pLOW-*smdAΔTMH*, ery^r	This study
IM377	NCTC8325-4, pLOW-*smdAΔTMH_mut1* (H145A), ery^r	This study
IM378	NCTC8325-4, pLOW-*smdAΔTMH_mut2* (R150A, T151A), ery^r	This study
IM379	NCTC8325-4, pLOW-*smdAΔTMH_mut3* (F280A, H281A), ery^r	This study
**Other strains**		
IM164	SH1000, pLOW-*smdA-flag*, ery^r	Lab collection

E. coli		
IM08B	DH10B, Δ*dcm*, P_{help}-*hsdMS*, P_{N25}-*hsdS* (expressing the *S. aureus* CC8 specific methylation genes)	(8)
BTH101	Used for BACTH analysis	Euromedex
XL1-Blue	Host strain	Agilent
**Strains harboring plasmids used to facilitate cloning**		
IM6	IM08B, pLOW-*ftsZ-m(sf)gfp*, amp^r	This study
IM98	IM08B, pLOW-*ftsZ-m(sf)gfp*_KpnI, amp^r	This study
IM33	IM08B, pLOW-*lacA-m(sf)gfp*, amp^r	This study
IM7	IM08B, pLOW-*ftsZ-mYFP*, amp^r	This study
IM8	IM08B, pLOW-*ftsZ-mKate2*, amp^r	This study
IM187	IM08B, pMAD- *smdA-flag_aad9*, amp^r	This study
**Strains used for BACTH assays**		
GS1225	XL1-Blue, pKNT25-*smdA*, kan^r	This study
GS1226	XL1-Blue, pUT18-*smdA*, amp^r	This study
GS1302	XL1-Blue, pKNT25-*smdAΔTMH*, kan^r	This study
GS1303	XL1-Blue, pUT18- *smdAΔTMH*, amp^r	This study
GS1134	XL1-Blue, pKT25-*pbp1*, kan^r	(6)
GS1135	XL1-Blue, pUT18C-*pbp1*, amp^r	(6)
GS1136	XL1-Blue, pKT25-*pbp2*, kan^r	(6)
GS1137	XL1-Blue, pUT18C-*pbp2*, amp^r	(6)
GS1138	XL1-Blue, pKT25-*pbp3*, kan^r	(6)
GS1139	XL1-Blue, pUT18C-*pbp3*, amp^r	(6)
GS1187	XL1-Blue, pKNT25-*ezrA*, kan^r	(6)
GS1188	XL1-Blue, pUT18-*ezrA*, amp^r	(6)

References

1. Novick R. 1967. Properties of a cryptic high-frequency transducing phage in *Staphylococcus aureus*. Virology 33:155-166.
2. Horsburgh MJ, Aish JL, White IJ, Shaw L, Lithgow JK, Foster SJ. 2002. σ^B modulates virulence determinant expression and stress resistance: characterization of a functional *rsbU* strain derived from *Staphylococcus aureus* 8325-4. J Bacteriol 184:5457-5467.
3. Kreiswirth BN, Löfdahl S, Betley MJ, O'reilly M, Schlievert PM, Bergdoll MS, Novick RP. 1983. The toxic shock syndrome exotoxin structural gene is not detectably transmitted by a prophage. Nature 305:709-712.
4. Herbert S, Ziebandt A-K, Ohlsen K, Schäfer T, Hecker M, Albrecht D, Novick R, Götz F. 2010. Repair of global regulators in *Staphylococcus aureus* 8325 and comparative analysis with other clinical isolates. Infect Immun 78:2877-2889.
5. Shafer WM, Iandolo JJ. 1979. Genetics of staphylococcal enterotoxin B in methicillin-resistant isolates of *Staphylococcus aureus*. Infect Immun 25:902-911.
6. Stamsås GA, Myrbråten IS, Straume D, Salehian Z, Veening JW, Håvarstein LS, Kjos M. 2018. CozEa and CozEb play overlapping and essential roles in controlling cell division in *Staphylococcus aureus*. Mol Microbiol 109:615-632.
7. Lund VA, Wacnik K, Turner RD, Cotterell BE, Walther CG, Fenn SJ, Grein F, Wollman AJ, Leake MC, Olivier N, Cadby A, Mesnage S, Jones S, Foster SJ. 2018. Molecular coordination of *Staphylococcus aureus* cell division. eLife 7:e32057.
8. Monk IR, Tree JJ, Howden BP, Stinear TP, Foster TJ. 2015. Complete bypass of restriction systems for major *Staphylococcus aureus* lineages. mBio 6:e00308-15.
