# Supplementary material for: SmdA is a Novel Cell Morphology Determinant in Staphylococcus aureus
Source: mBio. 2022 Mar 31;13(2):e03404-21. doi: 10.1128/mbio.03404-21 (PMC9040797; doi:10.1128/mbio.03404-21)
Supplement: TABLE S3 [file mbio.03404-21-st003.pdf]

Table S3. Primers used in this study.

Primer	Sequence (5'-3')[a]
**Primers used for construction of plasmids used in subcellular screening**	
im1_linker-FP_F_BamHI	ACTGGATCCCCGGATCTGGTGGAGAAGCTGCA
im2_m(sf)gfp_R_NotI_ EcoRI	AGTGAATTCGCGGCCGCTTACTTATAAAGCTCATCCATGCC
im77_SA1908_F_SalI_RBS	ATCGTCGAC*CAATAAAACTAGGAGGAAATTTAA*ATGGATTTATCTTCACCGATAG
im78_SA1908_R_BamHI	TCCGGGGATCCAATTGAATGATTCAATTTTATCCATC
**Primers used for making pLOW-*dcas9* compatible in MRSA**	
im183_pLOW_F	TACTGCAATCGGATGCGATTA
im184_pLOW_R	GTTAAGGGATGCATAAACTGC
im185_aad9_F_ol-im183	*TAATCGCATCCGATTGCAGTA*ATTGGGCCCACCTAGGATC
im186_aad9_R_ol-im184	*GCAGTTTATGCATCCCTTAAC*GCCGCGGTAATAAACTATCAA
**Primers used for construction of sgRNA plasmids used in depletion strains**	
mk299_sgRNA_1908	TACCTAAGGCAACTAAAAAA GTTTAAGAGCTATGCTGGAAACAG
mk323_sgRNA_01908_V2	ATAATGAGTCCAATGACTAT GTTTAAGAGCTATGCTGGAAACAG
**Primers used for chromosomal fusions and plasmids for localization studies**	
im147_SA1908_up_F_MluI	ACCTACGCGTGATTTTCGGTATATAAATGATAA
im148_SA1908_R_NotI_flag-overlap	*TCTTTATAATCAATATCATGATCTTTATAATCACCATCATGATCTTTATAATCC*GCGGCCGCATTGAATGATTCAATTTTATCCA
im149_aad9_up_F_SpeI_flag-overlap	*GATCATGATATTGATTATAAAGATGATGATGATAAATAA*ACTAGTATTGGGCCCACCTAGGAT
im150_aad9_down_R_1908 down-overlap	*TCATCACTTCAGCCTAACAT*CTCGAGGCCGCGGTAAT
im151_SA1908_down_F	ATGTTAGGCTGAAGTGATGA
im152_SA1908_down_R_BamHI	AGTCGGATCCTGATTTAAACCATCAATTCGC
im153_linker-FP_F_NotI	ACTGCGGCCGCCGGATCTGGTGGAGAAGCTG
im154_m(sf)gfp_R_SpeI	AGTACTAGTTTACTTATAAAGCTCATCCATG
im5_mKate_R_NotI_EcoRI	AGTGAATTCGCGGCCGCTTAACGGTGTCCCAATTTACTAGG
USHC109	GCGACGCGTTTAACGGTGTCCCAATTTACTAGG
USHC148	CGCGTCGACAGGAGGATAATTATTTATGTTAGAATTTGAACAAGG
im3_cfp_myfp_R_NotI_EcoRI	AGTGAATTCGCGGCCGCTTATTTATAAAGTTCGTCCATACC
**Primers used for verifying *smdA* silencing**	
im126_RT-q_pta_F	ATCATTGATGGCGAATTCCAAT
im127_RT-q_pta_R	GGACCAACTGCATCATATCC
im137_RT-q_SA1908_F	TATGTAACGGACATGAGATTAAT
im138_RT-q_SA1908_R	CTAATACCATTATAAACATGACC

**Primers used for construction of plasmids for overexpression**	
mk517_1908_R_NotI	ACGAGCGGCCGCCATATAGTCATCACTTCAGCCT
mk518_1908_F_RBS_SalI	ATCCAGTCGAC*CAATAAAACTAGGAGGAAATTTAA*ATG AGTAAGAAAAAAGTTAAGCGACAAAC
mk519_1908_H145A_F	GAACGAATTAGT**GC**TTTAGTATTAACAAG
mk520_1908_H145A_R	CTTGTTAATACTAAA**GC**ACTAATTCGTTC
mk521_1908_RT_AA_F	TTAGTATTAACA**GC**A**G**CTGGTCTTTATATT
mk522_1908_RT_AA_R	AATATAAAGACCAG**CT**G**C**TGTTAATACTAA
mk529_1908_FH_AA_F	CTTTAACAAATTTGTA**GC**C**GC**TGGTCGTATTCAAT
mk530_1908_FH_AA_R	ATTGAATACGACCA**GC**G**GC**TACAAATTTGTTAAAG
**Primers used for construction of plasmids to BACTH assays**	
gs718_SA1908_F_BamHI	GATCGGATCCCGATTTATCTTCACCGATAGTCATTG
gs719_SA1908_R_KpnI	GATCGGTACCCGATTGAATGATTCAATTTTATCCATC
gs735_SA1908ΔTMH_F_BamHI	GATCGGATCCCGGTAGTAAGAAAAAAGTTAAGCGAC

[a]Restriction sites are underlined, sequences included as overhang in italic and inserted mutations in bold.
